# Synthesis and characterization of semiaromatic copolyamide 10T/1014 with high performance and flexibility

**DOI:** 10.1080/15685551.2018.1446278

**Published:** 2018-03-12

**Authors:** Wutong Feng, Pingli Wang, Guangji Zou, Zhonglai Ren, Junhui Ji

**Affiliations:** a National Engineering Research Center of Engineering Plastics, Technical Institute of Physics and Chemistry, Chinese Academy of Sciences, Beijing, P.R. China; b College of Materials Science and Opto-Electronic Technology, University of Chinese Academy of Sciences, Beijing, P.R. China

**Keywords:** Semiaromatic polyamide, long chain, PA10T, heat resistance

## Abstract

Poly (decamethylene terephthalamide) (PA10T) is a kind of engineering plastics with high strength and high modulus, but one of its disadvantages is its low elongation at break. In order to improve the flexibility of PA10T, one aliphatic comonomer with a long alkyl chain is introduced to the molecular chain of PA10T. Then long chain semiaromatic copolyamides 10T/1014 were synthesized with different contents of 1014 units by polycondensation reaction of 1,10-diaminodecane, terephthalic acid and 1,12-dodecanedicarboxylic acid in deionized water. The intrinsic viscosities of the resultant polyamides ranged from 0.90 to 1.03 dL/g were obtained. The chemical and crystal structures of the copolymers were characterized by FTIR, ^1^H-NMR and WAXD. These copolyamides exhibited outstanding thermal properties with melting points range of 306–295 °C and degradation temperatures range of 479–472 °C at maximum degradation rate, and also have a wider processing window than PA10T. The tensile strength of PA10T/1014 copolymers decreased gradually from 80.02 to 72.95 MPa as the content of 1014 units increasing from 5 to 20 mol %, while the elongation at break increased significantly from 57 to 150%. The moisture content of 10T/1014 copolyamides decreased with increasing the 1014 unit contents. It suggests that 10T/1014 copolyamides could be a kind of promising heat-resistant engineering thermoplastic in the future applications.

## 1. Introduction

Aliphatic polyamides, such as nylon 6 and nylon 6,6, play an important role in automotive industry and electronic areas due to their excellent processability and toughness, but the lack of high thermal and dimensional stabilities limits their further application [1–6]. In contrast, wholly aromatic polyamides, such as poly (*m*-phenylene isophthalamide) (PMPI) and poly (*p*-phenylene terephthalamide) (PPTA), have been widely used in military and aeronautic fields for their outstanding heat resistance and mechanical property [7–12]. One drawback of most aramids is that their melting points are too high to be processed in a molten state. Recently, semiaromatic polyamides that combine good processability of aliphatic polyamides with the heat stability of wholly aromatic polyamides have been developed and attracted much attention [13–19]. The semiaromatic polyamide is one of the most promising classes of heat-resistant material that is able to be used above 150 °C for a long time.

As a kind of semiaromatic polyamide, poly (decamethylene terephthalamide) (PA10T) synthesized by polycondensation of 1,10-diaminodecane and terephthalic acid in organic solvent or deionized water has the properties of excellent heat resistance, good processability, outstanding dimensional stability, low water absorption and so on [20]. More importantly, 1,10-diaminodecane, one monomer of PA10T, is a renewable biomass energy sources and can be extracted from castor beans, which meets the requirements of green chemistry and sustainable development. Especially, oil resources are getting scarcer, and new bio-mass monomers or materials gradually play a key role in supporting the economic development. For example, with the appearance and wide application of surface-mount technology (SMT) that requires the melting point of polymers to be above 215 °C, PA10T has a high melting temperature (about 316 °C) and good heat-resistant properties, which can meet the requirement of SMT, and has a wide application in electronic, as well as automotive and aeronautic areas.

As mentioned above, the melting point of PA10T is as high as 316 °C, which is close to its beginning degradation temperature (350 °C) [21]. Normally, the processing methods of PA10T, like extrusion and injection, are both operated in the molten state, so just a narrow processing window is left between the melting and the degradation temperature of PA10T. In order to solve the problem, one feasible approach is to introduce a comonomer, especially an aliphatic comonomer to PA10T to form copolymers. As a result, a suitable content of comonomer can change the molecular regulation of PA10T and influence the crystallinity, which will decrease the melting temperature but still meet the requirement of thermo-stability which can be used for SMT technology. At the same time, some aliphatic segments copolymerized with PA10T can ameliorate the rigidity of molecular chains and increase the material flexibility. Basing on the idea, many copolyamides have been developed and researched [22–26]. By introducing aminoundecanoic acid as the comonomer, the melting point of PA10T/11 effectively decreased on the premise of ensuring the high heat resistant property [27]. When ω-laurolactame is copolymerized with polyamide 6, the target product PA6/12 has a higher elongation than PA6, which indicates that the flexible long aliphatic chains improve the ductility of copolymers [28]. Recently, long chain 1,12-dodecanedicarboxylic acid can be obtained by means of petroleum fermentation method using light wax oil as the raw material, and light wax oil is a kind of by-product of petroleum refining process and has a low price and abundant sources, which make it be a research hotspot [29]. And the structure of twelve methylene conjuncted together makes it easy to modify the conformation. Therefore, with long chain 1,12-dodecanedicarboxylic acid as the comonomer to modify PA10T, PA10T/1014 might have a lower melting temperature and be more flexible. At the same time, such a long carbon chain of the monomer will reduce the whole molecular density of amide bonds. As we all know, the density of amide bonds is a very important parameter for the properties of polyamide, like water absorption, crystallization and so on.

Aiming at studying the effects of 1,12-dodecanedicarboxylic acid as the comonomer on the properties of PA10T, a series of PA10T/1014 copolymers are synthesiezed from 1,10-diaminodecane, terephthalic acid and 1,12-dodecanedicarboxylic acid by polycondensation reaction. The influence of different contents of 1014 segments on the structure, crystallization, thermal stability, moisture content, and mechanical properties was studied by Fourier transform infrared (FTIR) spectroscopy, proton nuclear magnetic resonance (^1^H-NMR) spectroscopy, wide-angle X-ray scattering (WAXD), differential scanning calorimetry (DSC), thermogravimetric analysis (TGA), dynamic mechanical analysis (DMA), rapid halogen moisture meter, and tensile test, respectively. The research on the chemical modification of PA10T will be helpful for developing heat-resistant polyamides with expected performances from bio-based raw materials.

## 2. Experimental

### 2.1. Materials

1,10-diaminodecane (DMD) was purchased from Wuxi Yinda Co., Ltd. Terephthalic acid (PTA) was purchased from Jinan Mingxin Chemical Co., Ltd. 1,12-dodecanedicarboxylic acid (TDA) was purchased from Wuhan Yuancheng Group. Benzoic acid (BA) and sodium hypophosphite (SHP) were purchased from Shanghai Macklin Biochemical Co., Ltd. Concentrated sulfuric acid (96%) and N, N-dimethylformamide (DMF) were purchased from Beijing Chemical Reagent Company. All the reagents were used without future purification.

### 2.2. Synthesis

Monomers, sodium hypophosphite, benzoic acid and deionized water were added into an autoclave according to Table 1. The autoclave agitator was set to 50 rpm and the atmosphere was replaced by nitrogen for 10 times. Then the system was heated to 80 °C in 50 min and was held for about 1 h to get polyamide salts. Then the system was heated to 240 °C in 3 h and was held for about 30 min. During the heating process the pressure was controlled no more than 3 MPa by discharging steam. After that, water was removed from the autoclave to reduce the pressure to 0 MPa within 40 min, and meanwhile the system was kept at 240 °C. These pressure values that have deducted an atmospheric pressure were all read from the pressure gage of the autoclave. Solid prepolymers were obtained after the autoclave cooled to room temperature.

**Table 1.** Composition of PA10T and PA10T/1014 copolymers.

Run	Samples	DMD (mol)	PTA (mol)	TDA (mol)	BA (mol)	SHP (wt %)	H_2_O (wt %)
*a*	PA10T	1.01	1.00	–	0.02	0.1	150
*b*	PA10T/1014(5%)	1.01	0.95	0.05	0.02	0.1	150
*c*	PA10T/1014(10%)	1.01	0.90	0.10	0.02	0.1	150
*d*	PA10T/1014(15%)	1.01	0.85	0.15	0.02	0.1	150
*e*	PA10T/1014(20%)	1.01	0.80	0.20	0.02	0.1	150

The prepolymers were crushed and put into a vacuum dryer to increase viscosity. The vacuum dryer was heated to 260 °C and was held for about 4 h while keep the vacuum degree at about 50 Pa. Then the resultant white powders were dried for testing after washing repeatedly with DMF and deionized water.

### 2.3. Characterization

The samples were dissolved using 96% concentrated sulfuric acid to get 0.005 g/mL sulfuric acid solution of polymers used for viscosity measurements. Solutions containing a few visible gel particles passed through a G2 (10–15 μm) sand core funnel into the Ubbelohde viscometer and the measurement was conducted in a water bath at 25 ± 0.05 °C. Three parallel experiments were employed to measure the flow time, giving the statistic error bar less than ± 0.2 s. The flow time of the solution and concentrated sulfuric acid are recorded as *t*_1_ and *t*_0_, respectively. Relative viscosity recorded as *η*_r_ can be calculated by

(1) $$\eta_{r}=t_{1}/t_{0}$$

Specific viscosity recorded as *η*_sp_ can be obtained using

(2) $$\eta_{\mathrm{sp}}=t_{1}/t_{0}-1$$

Intrinsic viscosity recorded as [*η*] can be defined using the Solomon and Ciuta relationship [30], where *c* is the concentration of the solution:

(3) $$\left[\eta \right]\;=\;\left[2{\left(\eta_{\mathrm{sp}}-\;ln\eta_{r}\right)}^{1/2}\right]/c$$

The FTIR measurements were carried out on a JASCO FT/IR-6800 instrument in the range of 4000–600 cm^−1^.

The ^1^H-NMR spectra were collected on a BRUKER AVANCE Ⅱ-400 apparatus. The samples were dissolved in trifluoroacetic acid-D (CF_3_COOD).

Diffraction patterns were obtained with a BRUKER D8 focus diffractometer. Data was collected in 2θ range of 5° to 65° with an increment of 0.02° and a scan speed of 0.1 s/step.

Melting temperatures and crystallization temperatures of the samples were obtained with a METTLER DSC1 instrument. The sample sizes ranged from 4 to 8 mg and the measurement was carried out under a nitrogen flow of 50 mL/min. The measurement consists of three stages. In the first stage the sample was heated to 340 °C at a constant rate of 20 °C/min and was kept at 340 °C for 5 min to eliminate thermal history. Then the sample was cooled to 0 °C at a rate of 10 °C/min to get the cooling curve. In the last stage, the sample was heated to 340 °C again with a 10 °C/min heating rate. Data was collected from the cooling and the second-heating process.

The TGA measurement was performed using a TA Q50 thermal analyzer with a heating rate of 10 °C/min from 0 to 600 °C in a nitrogen stream. The sample sizes were about 5 mg and placed in a platinum pan for TGA measurement.

The DMA measurement was carried out on a METTLER DMA/SDTA 861e apparatus in the tension mode at 1 Hz and a heating rate of 3 °C/min from −30 to 200 °C. The force amplitude was 5 N and the displacement amplitude was 15 μm. The rectangle samples of 18 × 4.5 × 1 mm^3^ were prepared by injection molding with HAAKE Mini Jet Pro.

Tensile tests were done with an INSTRON-5699 instrument according to GB/T 2918 (China standard). The dumbbell-shaped specimens of 25 ± 0.25 mm length, 4 mm width, and 2 mm thickness were made by injection moulding with HAAKE Mini Jet Pro at 340 °C. The samples were stretched at 25 °C with a 5 mm/min stretching rate.

The moisture content was obtained with the Rapid Halogen Moisture Meter VM-15. The samples were about 3 g and the test temperature was 120 °C.

## 3. Results and discussion

### 3.1. Synthesis of PA10T and PA10T/1014

**Scheme 1.**
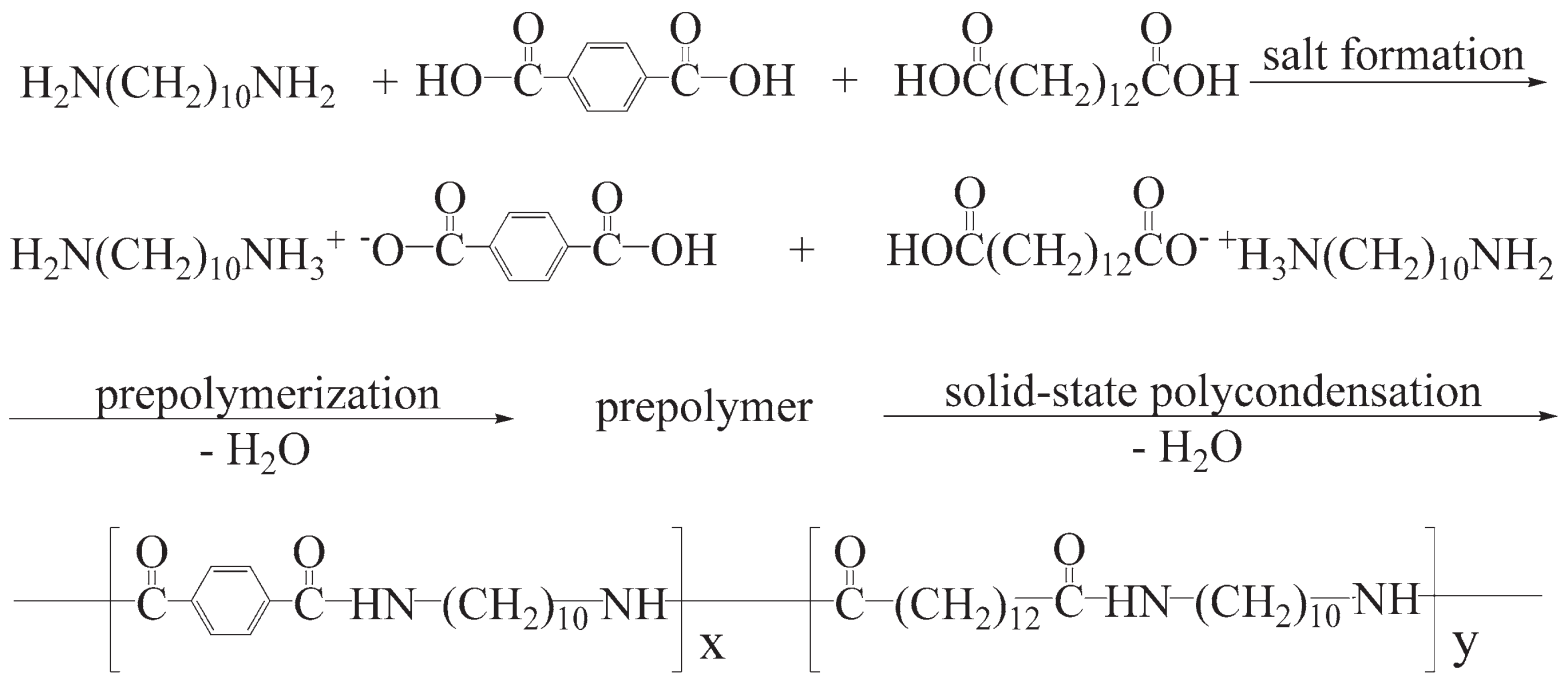
Synthesis route of PA10T/1014 copolymers, where *x* and *y* are variable.

PA10T and PA10T/1014 prepolymers were prepared from 1,10-diaminodecane, terephthalic acid and 1,12-dodecanedicarboxylic acid by polycondensation reaction as shown in Scheme 1. In the process of pre-polymerization, the system was kept at 80 °C for about 1 h to get polyamide salts, which is to reduce the volatilization loss of 1,10-diaminodecane during the process of discharging steam. The synthesis procedure is one-step polycondensation that omitted the procedure of separating 10T salt, which greatly simplified the experimental procedure and reduced the cost of production compared with traditional methods of salt formation, prepolymerization and solid-polymerization using dimethylformamide as the solvent [31]. The important problem was to make sure an accurate equivalent ratio of acid to amine if polyamide-salt was not separated and refined. Therefore, a slight excess of 1 mol % DMD was added to compensate the volatilization of DMD, which could ensure PTA completely react with DMD.

In the initial stage of prepolymerization, 150 wt % deionized water was added into the autoclave acting as a reaction medium to make the reactants well mixed and contacted. And the procedure of salt formation is conducted gradually and slowly because the water solubility of DMD and PTA is very small and this is a cyclic process of dissolving-salt forming-separating out step by step. The high vapor pressure of the reaction is supplied by water. Meanwhile as the byproduct, water has a retarding effect on the polymerization process in which the stage of discharging steam is particularly important. Postpolycondensation is a stage of solid-state polycondensation (SSP) in which maintaining high vacuum is necessary for avoiding side reaction [32].

### 3.2. Intrinsic viscosity

Intrinsic viscosities are used to estimate the relative number average molecular weights of copolymers with Mark–Houwink equation

(4) $$\left[\eta \right]\;=KM_{\eta }^{a}$$

which presents the relationship between [*η*] and *M*_η_. However, it has not been reported that the Mark-Houwink equation constants *K* and *α* for PA10T or PA10T/1014. The equation

(5) $$\left[\eta \right]=0.000558\left(\text{dL}/\text{g}\right){\left(M_{\eta }\right)}^{\wedge }0.81$$

had been used to estimate *M*_η_ of PA10T/6T and PA10T/12T copolymers [33]. Because the chemical structure of PA10T/1014 is similar with PA10T/6T, it is reasonable to use the equation to estimate *M*_η_ of PA10T and PA10T/1014 copolymers and the data is listed in Table 2. The intrinsic viscosities of PA10T and PA10T/1014 copolymers were ranged from 0.90 to 1.03 dL/g and the *M*_η_ values of the resultant samples were calculated and ranged from 9120 to 10,773, which proves that polymers with high number-average molecular weight were synthesized. Meanwhile, it could be concluded that *M*_η_ values of PA10T and PA10T/1014 lied almost at the same level, which is helpful to analyse the influence of 1014 segments on the properties of PA10T/1014 copolymers.

**Table 2.** Intrinsic viscosities and estimated *M*_η_ values of PA10T and PA10T/1014 copolymers.

Samples	*a*	*b*	*c*	*d*	*e*
[*η*] (dL/g)	1.03	0.99	0.90	0.92	0.96
*M*_η_ (g/mol)	10,773	10,259	9,120	9,371	9,877

### 3.3. FTIR characterization

**Figure 1.**
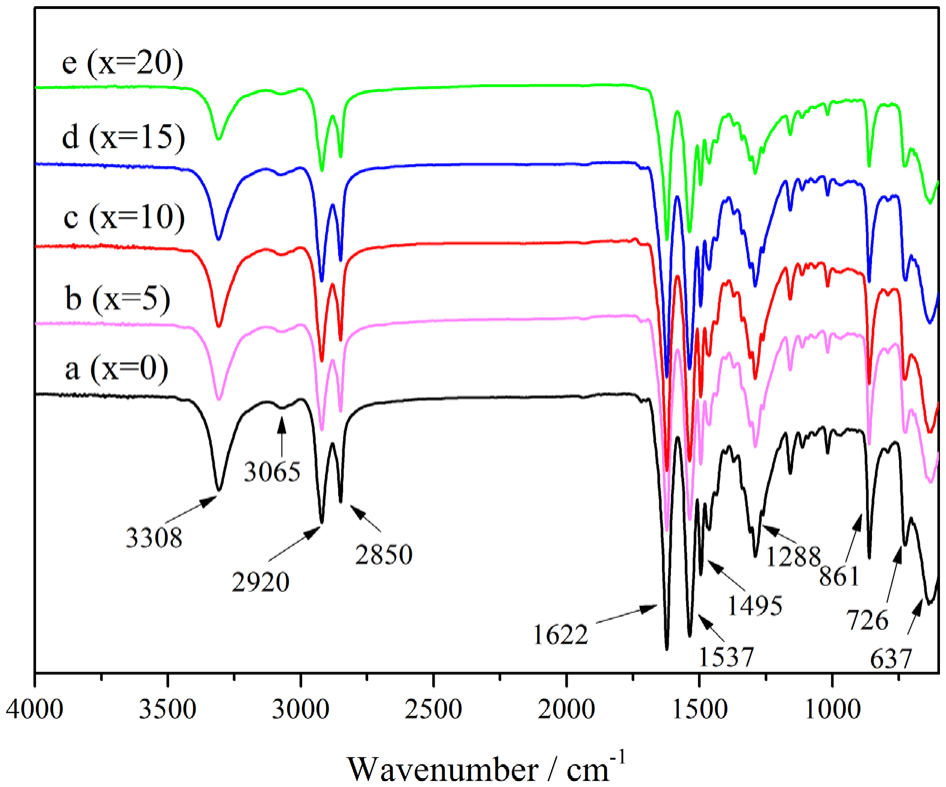
FTIR spectra of PA10T/1014(*x*%) copolymers.

FTIR spectra of PA10T and PA10T/1014(*x*%) copolymers are shown in Figure 1. PA10T/1014 copolymers exhibit almost the same infrared spectra characteristics as PA10T. The band at 3308 cm^−1^ is assigned to hydrogen bond and ν_N–H_ in polyamide. The bands at 1622, 1537, 1495, 1288 and 637 cm^−1^ are assigned to amide Ⅰ (*ν*_C=O_), amide Ⅱ (*ν*_C–N_ and *δ*_N–H_), amide Ⅲ (*ν*_C–N_ and *δ*_C–H_), amide Ⅳ (*ν*_C–CO_) and amide Ⅴ (*δ*_N–H_), respectively [34]. And the intensity of these bands decrease with the content of 1014 units increasing, which attributed to the longer molecular chain of 1,12-dodecanedicarboxylic acid compared with terephthalic acid so that the amide bonds density of PA10T/1014 is lower than PA10T. The band at 3065 cm^−1^ could be attributed to combined frequency-doubling absorption peak of *ν*_C–N_ and *ν*_N–H_. All above characteristic bands of amide bond suggest that desirable PA10T and PA10T/1014 copolymers were successfully synthesized.

#### 3.4. ^**1**^H-NMR  characterization

**Figure 2.**
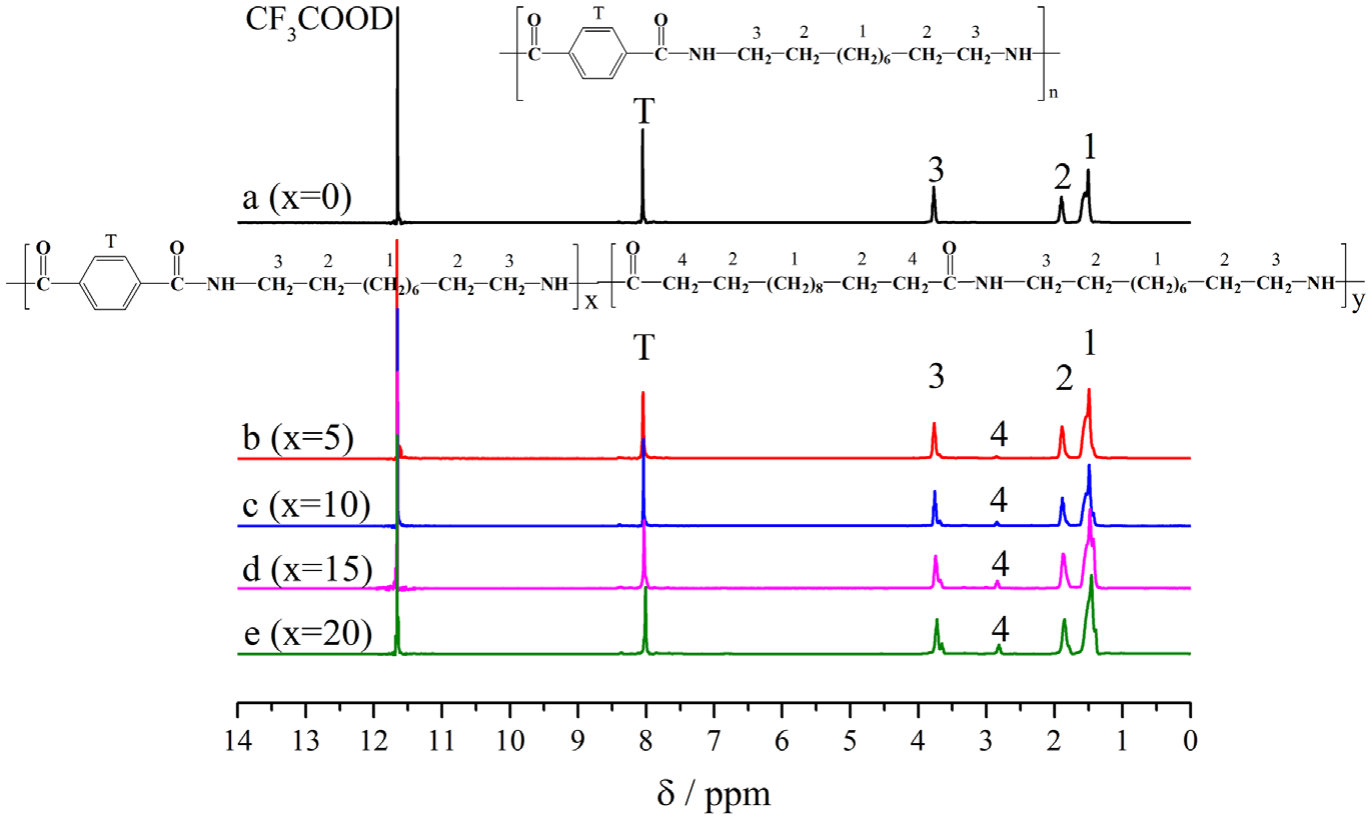
^1^H-NMR spectra of PA10T/1014(*x*%) copolymers.

^1^H-NMR spectroscopy could be used to determine copolymer chemical structure and composition. As demonstrated in Figure 2, the chemical shift at 11.615 ppm is attributed to the solvent peaks of trifluoroacetic acid-D. The chemical shifts at 1.430 ppm, 1.826, 3.700 and 7.980 ppm are attributed to the proton signals at position 1, 2, 3, and T, respectively. And they all belong to PA10T units. The integral area ratio of *δ*_1_, *δ*_2_, *δ*_3_ and *δ*_T_ is close to 3:1:1:1 for PA10T in Table 3(a), which agrees with the expected molecular structure of pure PA10T. For PA10T/1014 copolymer, the CH_2_ of 1,12-dodecanedicarboxylic acid attached to the amide groups presented at 2.788 ppm (position 4). Consequently, the mole contents of PA1014 segments were calculated according to the equation *δ*_4_/*δ*_3._ Here, *δ*_4_ and *δ*_3_ designated the intensities of peak 3 and peak 4. As listed in Table 3, the actual compositions of PA1014 segments in the copolymers are 5, 11, 15 and 20%, which are agreed well with the contents of PA1014 in feed ratio.

**Table 3.** ^1^H-NMR integral data of PA10T and PA10T/1014 copolymers.

Samples	*δ*_1_	*δ*_2_	*δ*_3_	*δ*_4_	*δ*_T_	*n*_TDA_/*n*_DMD_ (actual value) (%)	*δ*_4_/*δ*_3_ (calculated value) (%)
*a*	2.99	0.97	1.00	–	0.99	–	–
*b*	3.20	1.06	1.00	0.05	0.94	4.95	5
*c*	3.43	1.12	1.00	0.11	0.90	9.90	11
*d*	3.64	1.15	1.00	0.15	0.84	14.85	15
*e*	3.85	1.21	1.00	0.20	0.79	19.80	20

### 3.5. WAXD analysis

**Figure 3.**
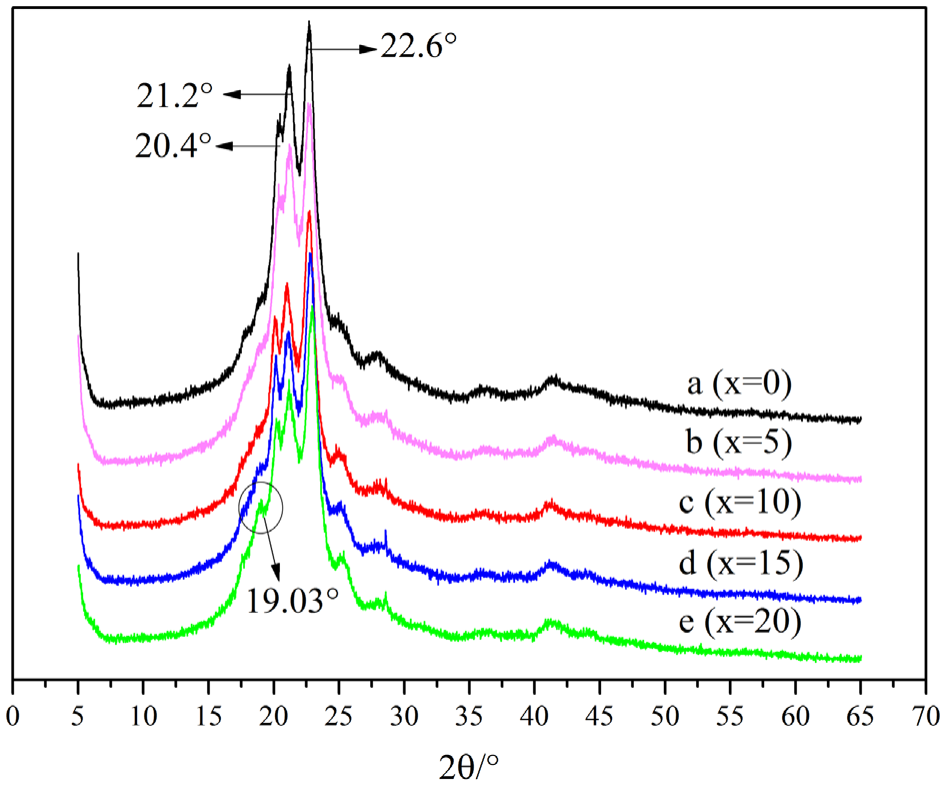
XRD curves of PA10T/1014(*x*%) copolymers.

X-ray diffraction patterns of PA10T/1014 are given in Figure 3, which could be used to investigate the crystallization of PA10T/1014 copolymers. Diffraction patterns of PA10T show sharp diffraction peaks at approximately 22.6°, 21.2° and 20.4°. For PA10T/1014 copolymers, they were found to show the similar diffraction peaks assignable to those of PA10T, which indicated that the copolymerization of 1,12-dodecanedicarboxylic acid did not change the crystal structures of PA10T segments. However, it forms a new weak peak at 19.03° as the content of 1014 units increased especially when the content is 20 mol %, which can be assigned to the α-form crystal of PA1014 segments [35]. That is, PA1014 segments could form crystals themselves if the content of PA1014 is enough.

### 3.6. DSC analysis

**Figure 4.**
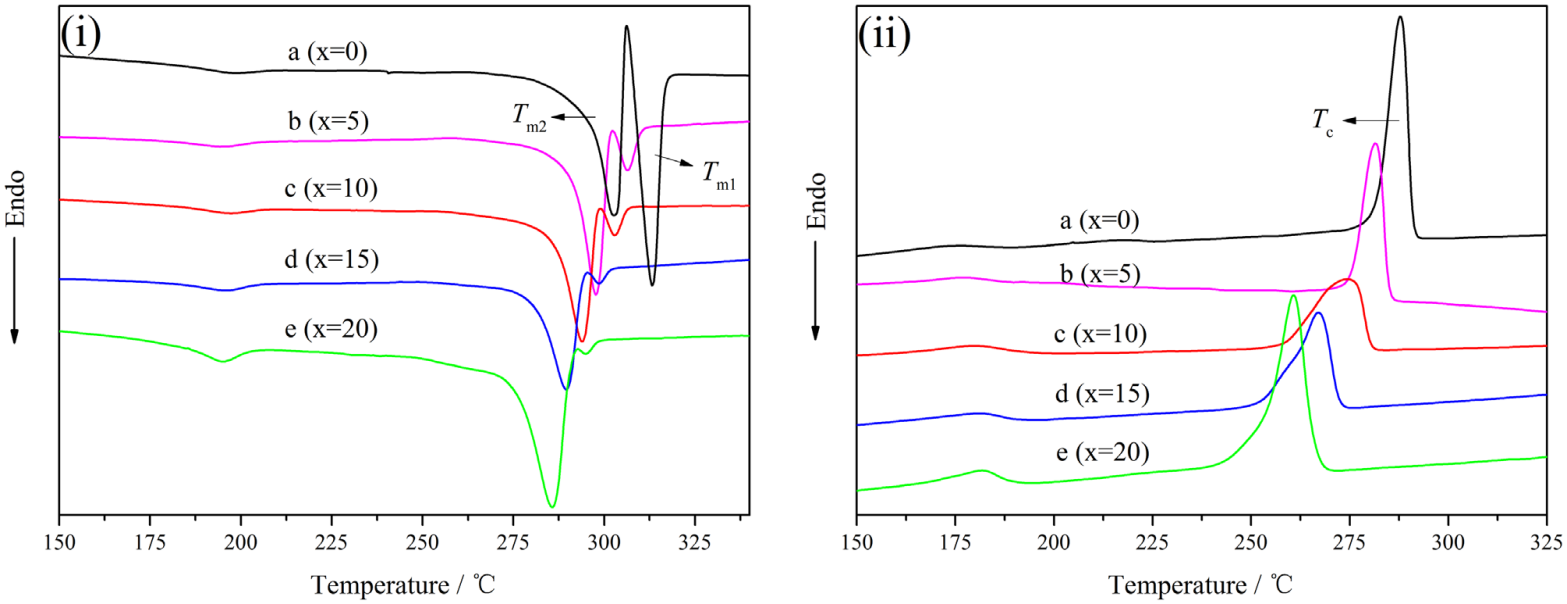
DSC curves of PA10T/1014(*x*%) copolymers: (i) heating and (ii) cooling curves.

**Figure 5.**
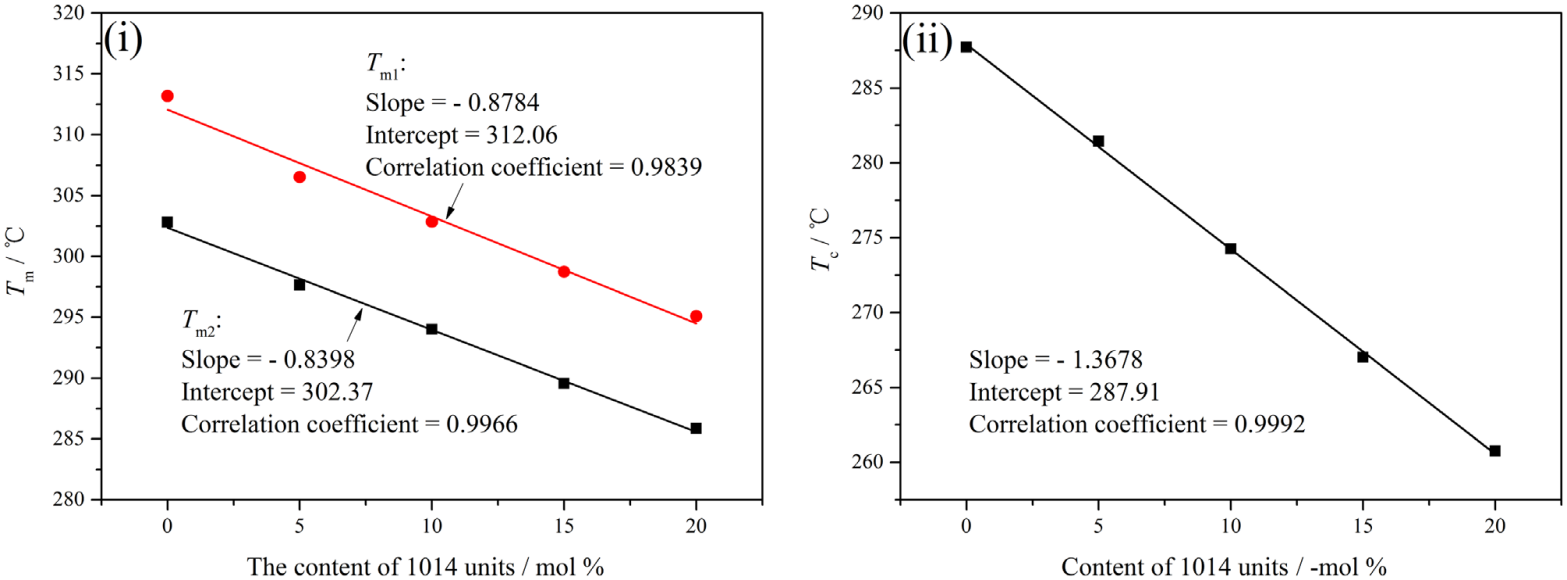
Linear relations between (i) *T*_m_, (ii) *T*_c_ and the content of 1014 units.

The thermal properties and melt crystallization behaviors of the copolymers have direct influence on their using and processing performance, which were characterized by DSC as depicted in Figure 4. First, the melting curves of PA10T and PA10T/1014 copolymers exhibit obvious double-melting endotherms which are a common phenomenon in semi-crystalline polymers, and the double melting behavior might be attributed to recrystallization phenomenon [36]. Accompany with the increasing of 1014 units, both *T*_m1_ and *T*_m2_ shifted to low temperature. Because the polymer chain became more flexible with the copolymerization of aliphatic 1014 segments, the melting temperature of PA10T/1014 was lower than PA10T. At the same time, the shape of double endothermic peaks varied. The endothermic peak at *T*_m1_ gradually diminished, while the peak at *T*_m2_ enlarged. As more and more aliphatic 1014 units were induced to the molecular chain of PA10T, the randomness of the resulting copolymer would lead to less recrystallization. Second, the crystallization temperatures of PA10T/1014 copolymers decrease with the content of 1014 units increasing. Though the flexibility of aliphatic 1014 segments benefits the adjustment of molecular conformation, lower hydrogen bond density and regularity would inhibit the crystallization of copolymers. As a conclusion, the crystallization temperature shifted to low temperature, and the degree of crystallinity decreased, which was proved by the value of melting enthalpy in Table 4. Third, in order to further investigate the effect of molecular composition on the crystallization, a quantitative analysis was carried out as presented in Figure 5. Both *T*_m_ and *T*_c_ had a linear relationship with the content of 1014 segments, which is a guide to design and synthesize new PA10T/1014 copolymers of given thermal properties by adjusting the ratio of 1014 units to 10T units.

**Table 4.** The thermal property data of PA10T/1014(*x*%) copolymers.

Samples	*a*(*x* = 0)	*b*(*x* = 5)	*c*(*x* = 10)	*d*(*x* = 15)	*e*(*x* = 20)
*T*_m1_ (°C)	313.18	306.52	302.85	298.74	295.11
*T*_m2_ (°C)	302.80	297.64	294.00	289.55	285.85
∆*H*_m_ (J/g)	−100.31	−58.44	−52.55	−42.25	−29.94
*T*_c_ (°C)	287.73	281.43	274.25	267.00	260.75
*T*_max_ (°C)	479.39	478.76	478.32	474.16	472.45
*T*_5%_ (°C)	435.79	430.54	428.85	426.34	424.22
∆*T* (°C)	122.61	124.02	126.00	127.60	129.11
*T*_g_ (°C)	132.31	122.78	111.49	105.26	94.86

### 3.7. DMA analysis

**Figure 6.**
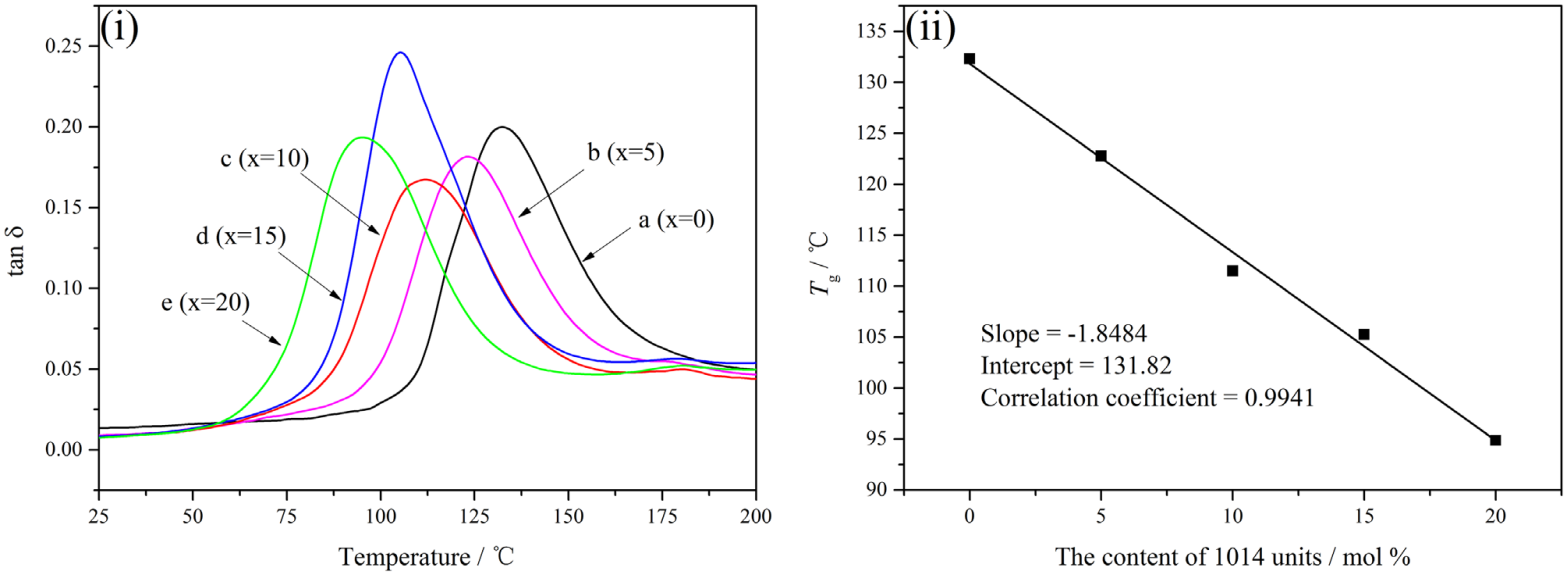
(i) DMA curves of PA10T/1014(*x*%) copolymers, and (ii) linear relation between *T*_g_ and the content of 1014 units.

Dynamic thermomechanical analysis (DMA) is an important and effective method to characterize the thermomechanical properties. And the glass transition temperature (*T*_g_), as determined by the tan δ maximum, has an intimate relationship with the composition and comonomer sequence of copolymer, which is a macroscopic express of inner molecules changing between moving and freezing. As shown in Figure 6(i), *T*_g_ of PA10T is 132.31 °C and is higher than aliphatic polyamides, for instance, *T*_g_ of PA6 is only 58.7 °C [37]. The molecular structure of PA10T contains rigid benzene rings which limit the appearance of more possible molecular chain conformation to reduce the flexibility of polymer chain. So more energy is consumed to move the molecular chain from a frozen state, which makes PA10T have a higher glass transition temperature. When more and more 1014 units are introduced to PA10T, the reduction of rigid benzene rings and hydrogen bonds decreased *T*_*g*_ of PA10T/1014. Moreover, the relationship between *T*_g_ and the content of 1014 units is a linear dependence as shown in Figure 6(ii). The slope of best fitting straight line is −1.8484, which means the introduction of 1014 units can decrease the *T*_g_ of PA10T evidently. Combining the linear relation between *T*_m_ and the content of 1014 units, the temperature interval between *T*_m_ and *T*_g_ increases gradually with the content of 1014 units increasing, which can broaden the using temperature range of the materials.

### 3.8. TGA characterization

**Figure 7.**
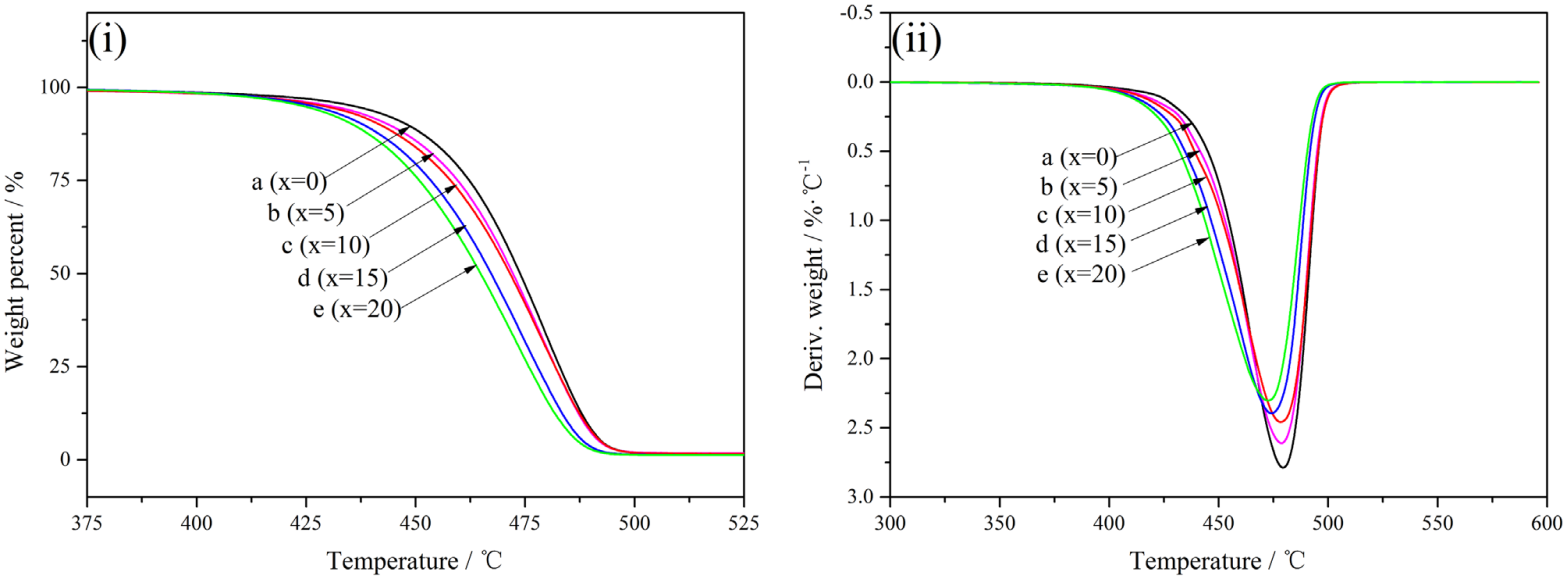
(i) TG and (ii) DTG curves of PA10T/1014(*x*%) copolymers.

The thermal stability of PA10T and PA10T/1014 copolymers was studied by TG and DTG, as depicted in Figure 7. All the curves of PA10T and PA10T/1014 copolymers show one stage of weight loss, which does accord with the mechanism of random chain scission on thermal degradation of polymers [27]. The temperature of maximum peak (*T*_max_) that is referred to the temperature at the maximum degradation rate can be obtained from the DTG curves, as shown in Figure 7(ii). *T*_max_ of PA10T is 479.39 °C. With the content of 1014 units increasing from 5 to 20%, *T*_max_ of PA10T/1014 copolymers decreases gradually from 478.76 °C to 473.94 °C, which is attributed to the increase of flexible aliphatic segments. Although the degradation temperatures of PA10T/1014 copolymers decrease to different extent, they are still higher than aliphatic polyamides [38]. Normally, the onset degradation temperatures (*T*_5%_) of PA66 and PA46 are 402.9 and 395.2 °C [39–42], respectively, so PA10T/1014 copolymers still have remarkable heat-resistant properties. As described above, the processability of polyamide is important for the usage, and a narrow processing window restricts the use of PA10T. The extrusion, injection molding and other molten process of PA10T are carried out above its melting temperature, so we define the processing window (∆*T*) as the temperature interval between *T*_5%_ and *T*_m1_. As shown in Table 4, the processing window is found to increase with the content of 1014 units increasing. ∆*T* was about 122.61 °C for PA10T, but the value was 129.11 °C with 20 mol % PA1014 segments, which has been broadened about 7 °C. These results indicated that the copolymerization with 1,12-dodecanedicarboxylic acid could optimized the processability of PA10T and the PA10T/1014 copolymer still had excellent thermal stability.

### 3.9. Mechanical properties

**Figure 8.**
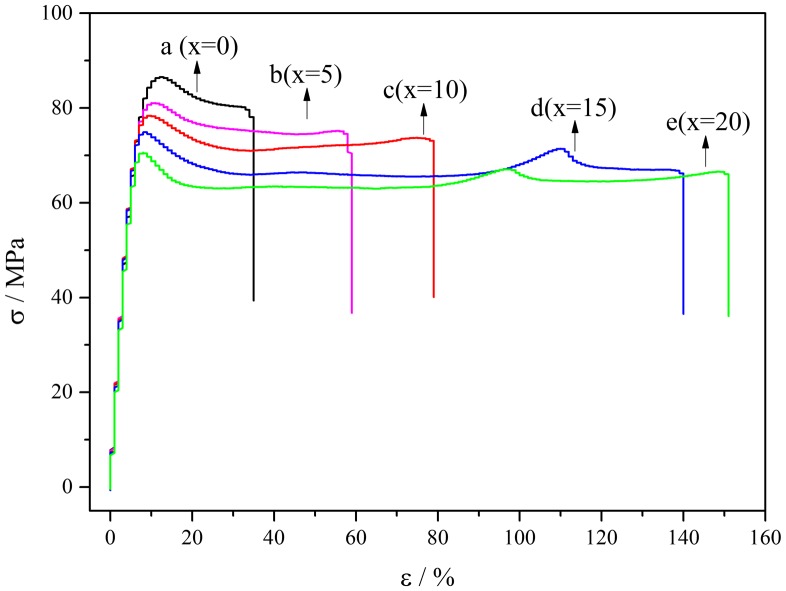
Stress-strain curves of PA10T/1014(*x*%) copolymers.

**Figure 9.**
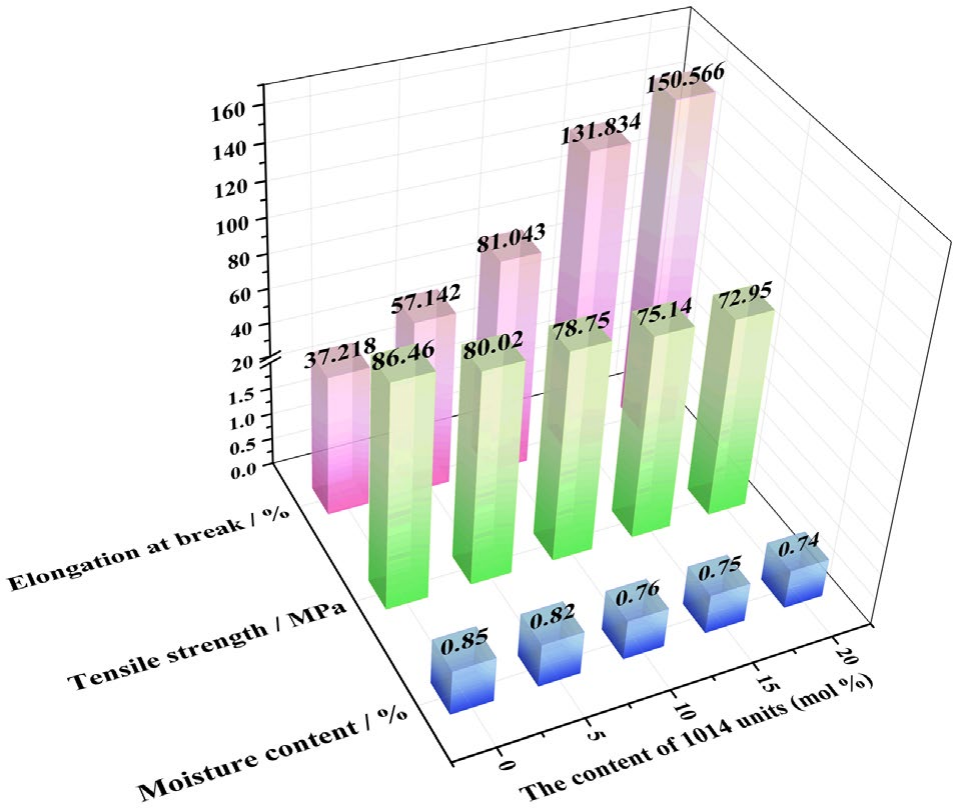
The mechanical properties and moisture content of PA10T/1014(*x*%) copolymers.

PA10T has good tensile strength, but the ductility and flexibility are weak. The purpose of adding aliphatic 1014 units is to improve the ductility of PA10T, and the mechanical properties of PA10T/1014 copolymer depended mainly on the composition of copolymer and the crystallization of the materials. Hence, the effect of different contents of 1014 segments on their mechanical properties was checked by tensile measurements. As indicated in Figure 8 and Figure 9, a relatively sharp yield is observed and corresponding yield stress is about 88 MPa for PA10T. After a slight drop, the stress nearly unchanged and lied at a plateau region until break, which is a typical tensile behavior of crystalline polymer. And the fracture stress is about 80.02 MPa, while the elongation at break is 37.218%. For PA10T/1014 copolymer, the stress-strain curves showed a similar shape, and the yield strength gradually dropped with increasing the content of 1014 segments. For example, the tensile strength of PA10T/1014 (20%) is 72.95 MPa, down by 10%. However, an outstanding elongation at break was observed with the increase of 1014 contents. The elongation at break of PA10T/1014 copolymers are 57.142, 81.043, 131.834 and 150.566% when the content of 1014 units are 5 mol %, 10 mol %, 15 mol % and 20 mol %, respectively. Compared with PA10T, the elongation at break of PA10T/1014 (20%) had an enhancement of 300%, which was due to the better flexibility of aliphatic PA1014 chains. The molecular chain of PA10T/1014 copolymers is more flexible and the chain mobility has been increased because some rigid benzene rings were substituted by aliphatic chains. It was reasonable that the tensile strength appeared slight drop with the junction of 1014 segments. Besides, the reduction of crystallinity in accordance with DSC results could cause the decrease of the tensile strength [28]. However, the slight loss of strength is in exchange for a multiply increase in elongation at break. It can be said that PA10T/1014 copolymers have outstanding ductility under stretching conditions.

In addition, the melting temperature of PA9T is about 308 °C [14,43], which is almost the same with PA10T/1014 with 5% 1014 segment. However, the elongation at break of PA10T/1014 (5%) is much higher than PA9T, which is just 20% [23]. This indicated that the material of PA10T modified with 1,12-dodecanedicarboxylic acid as the comonomer has a more outstanding ductility than PA9T, though they possess similar heat-resistant properties.

### 3.10. Moisture content

Moisture content is usually used to characterize the hygroscopicity of materials, that is, the property of materials absorbing moisture in moist air, which is an important parameter for the usage of polyamide. In fact, the moisture content in the air can be used to evaluate the water absorption of polyamide. As shown in Figure 9, the moisture content of PA10T is 0.85%, and the moisture content of PA10T/1014 copolymers decreased with the content of 1014 units increasing. The amide bond on the polymer backbone is a hydrophilic group, which is the main water absorbing group of polyamide. Compared to PA10T, PA10T/1014 copolymers have lower amide bond density. As more and more terephthalic acid were replaced by 1,12-dodecanedicarboxylic acid, the amide bond density of copolymers got lower and lower.

## 4. Conclusions

Semiaromatic PA10T/1014 copolymers were successfully prepared through polycondensation reaction under high temperature and pressure. FTIR and ^1^H-NMR spectra confirmed the chemical structure and X-ray diffraction patterns characterized the crystal structure. The *T*_m_, *T*_c_, *T*_g_ and *T*_d_ were obtained by means of DSC, DMA and TGA, and all the values decreased with increasing the content of 1014 units, but the material’s processing window and using temperature range were broadened. Comparing with the PA10T, the tensile strength of PA10T/1014 copolymers decreased gradually from 80.02 to 72.95 MPa with the content of 1014 units increasing from 5 to 20 mol %, while the elongation at break increased obviously from 57 to 150%. And 10T/1014 copolyamides have lower moisture content than PA10T. To summarize, 10T/1014 copolyamides are a kind of heat-resistant engineering plastics with high elongation at break and low moisture content, which might have more applications in many fields.

## Disclosure statement

No potential conflict of interest was reported by the authors.

## Funding

This work was supported by the National Natural Science Foundation of China [grant number 51473175].
